# Measuring personal recovery in a low-intensity community mental healthcare setting: validation of the Dutch version of the individual recovery outcomes counter (I.ROC)

**DOI:** 10.1186/s12888-022-03697-6

**Published:** 2022-01-14

**Authors:** Thijs Beckers, Bauke Koekkoek, Giel Hutschemaekers, Bridey Rudd, Bea Tiemens

**Affiliations:** 1 Research Group Social Psychiatry and Mental Health Nursing, Hogeschool Van Arnhem Nijmegen (University of Applied Sciences), Nijmegen, Netherlands; 2 Primary Mental Healthcare, MET ggz, Roermond, Netherlands; 3 Clinical Psychology, Behavioural Science Institute, Radboud University, Nijmegen, Netherlands; 4 Pro Persona Research, Wolfheze, Netherlands; 5 Indigo Service Organization, Utrecht, Netherlands; 6 Penumbra, Edinburgh, United Kingdom; 7 Department of Psychology, Abertay University, Dundee, Scotland

**Keywords:** I.ROC, Recovery, Psychometric evaluation, Translation

## Abstract

**Background:**

Measuring progress in treatment is essential for systematic evaluation by service users and their care providers. In low-intensity community mental healthcare, a questionnaire to measure progress in treatment should be aimed at personal recovery and should require little effort to complete.

**Methods:**

The Individual Recovery Outcome Counter (I.ROC) was translated from English into Dutch, and psychometric evaluations were performed. Data were collected on personal recovery (Recovery Assessment Scale), quality of life (Manchester Short Assessment of Quality of Life), and symptoms of mental illness and social functioning (Outcome Questionnaire, OQ-45) for assessing the validity of the I.ROC. Test–retest reliability was evaluated by calculating the Intraclass Correlation Coefficient and internal consistency was evaluated by calculating Cronbach’s alpha. Exploratory factor analysis was performed to determine construct validity. To assess convergent validity, the I.ROC was compared to relevant questionnaires by calculating Pearson correlation coefficients. To evaluate discriminant validity, I.ROC scores of certain subgroups were compared using either a t-test or analysis of variance.

**Results:**

There were 764 participants in this study who mostly completed more than one I.ROC (total *n* = 2,863). The I.ROC aimed to measure the concept of personal recovery as a whole, which was confirmed by a factor analysis. The test–retest reliability was satisfactory (Intraclass Correlation Coefficient is 0.856), as were the internal consistency (Cronbachs Alpha is 0.921) and the convergent validity. Sensitivity to change was small, but comparable to that of the OQ-45.

**Conclusions:**

The Dutch version of the I.ROC appears to have satisfactory psychometric properties to warrant its use in daily practice. Discriminant validity and sensitivity to change need further research.

## Introduction

Measuring progress in treatment is an essential part of evidence-based practice. Healthcare professionals struggle to reliably evaluate service users’ progress within individual treatments [1]. Using reliable and validated questionnaires improves the quality of such an evaluation [2]. Additionally, when using questionnaires for treatment evaluation, healthcare providers can notice lack of progress early in the treatment process [3–5].

Traditionally, most treatments have aimed at reducing symptoms of the mental illness; this is often referred to as clinical recovery [6, 7]. More recently, the focus of treatment has expanded—initially to include rehabilitation, and then further broadened to reflect social and personal recovery outcomes, although much work has to be done to improve the focus on personal recovery in daily practice [8]. Social recovery encompasses the position the person occupies in society, regarding, for example, paid employment, financial situation, and social network [9]. Social recovery also is an element of personal recovery, which Anthony [10] defined *personal recovery* as “a deeply personal, unique process of changing one’s attitudes, values, feelings, goals, skills, and/or roles. It is a way of living a satisfying, hopeful, and contributing life, even within the limitations caused by illness. (p527)” The main components of personal recovery are described in the CHIME framework as connectedness, hope, and optimism about the future, one’s identity, meaning in life, and empowerment [11, 12]. Stuart et al. added difficulties (i.e. psychological difficulties or mental illness) to the CHIME framework and renamed it to CHIME-D [13]. Given this expansion and personalization of the goals of treatment for mental illness, questionnaires intended for use as a routine outcome-monitoring tool should reflect the changes that are described in the objectives of the care. Such instruments should additionally (a) have a broad focus, leaving room for individual goals, (b) include, but not focus exclusively on, clinical recovery, and (c) be completed by the person with a severe mental illness instead of the service provider.

## Background

In the Netherlands, the care for people with severe mental illness is commonly provided by community mental healthcare teams, such as Flexible Assertive Community Treatment teams [14]. Community mental healthcare, however, is being increasingly provided by (advanced practice) nurses or psychologists in cooperation with primary care physicians. Such low-intensity community mental healthcare usually consists of a session with a mental health nurse or a psychologist every four-to-eight weeks in addition to primary medical care provided by a primary care physician [15].

For routine assessment to be effective, a questionnaire should be administered frequently. This need is, however, currently not being fulfilled because questionnaires aimed at personal recovery commonly have between 10 and 50 questions (most often 20 to 40) and often require a considerable time investment to complete [16]. Furthermore, there are additional requirements for a questionnaire to measure personal recovery in low-intensity community mental healthcare. The first requirement is that the questionnaire needs to be completed by the service user (possibly aided by a healthcare professional). Because, however, personal recovery is a unique and personal process, it cannot be judged by a healthcare professional. Secondly, the psychometric properties of the questionnaire should be assessed sufficiently, which is not the case for most personal recovery questionnaires [17, 18]. Thirdly, in order to promote good response rates and meaningful use of recovery measures, questionnaires should appeal to the service user in terms of both content and format (illustrated and with short, clear questions instead of longer questions in black on white text).

**Fig. 1:**
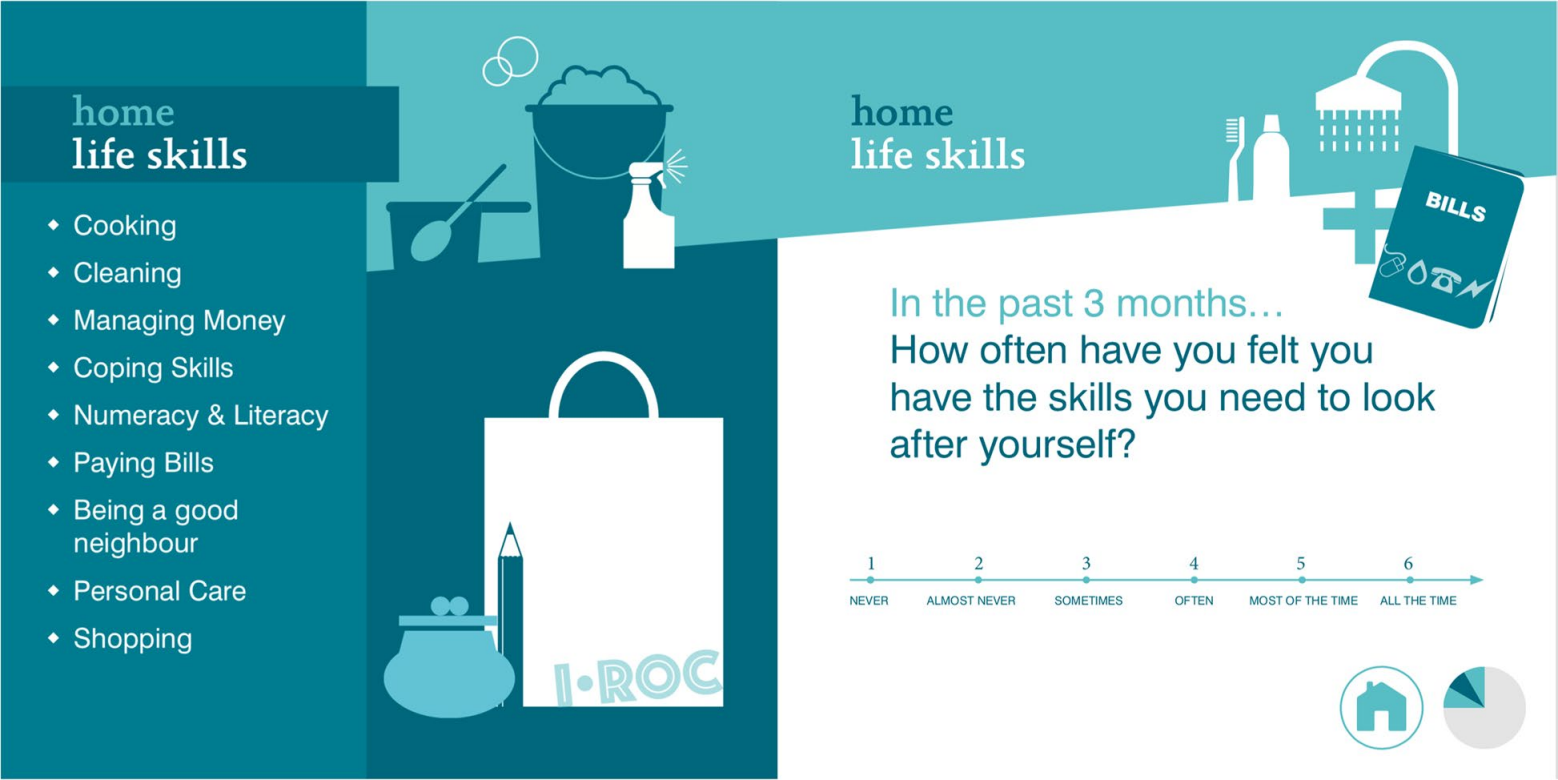
Example question on the I.ROC

One questionnaire that meets all of the requirements stated above is the Individual Recovery Outcome Counter (I.ROC). The I.ROC was developed in Scotland in 2011 for use with people with social or mental health problems (both mild and severe problems). The I.ROC was developed in close collaboration with practitioners and people with lived experiences of mental illness. It is a guided self-assessment tool for measuring personal recovery, social recovery, and symptomatic recovery [19]. The I.ROC consists of 12 questions, which are divided into four groups: home, opportunity, people, and empowerment. Each question is placed on an illustrated page and is accompanied by a list of 8-to-12 keywords for clarification and a 6-point Likert scale for answering the question (see Fig. 1). Individual questions address topics such as *life skills*, *safety and comfort*, *personal network*, and *valuing myself,* and they converge with the CHIME-D framework discussed earlier. The English version of the I.ROC has been found to be a valid and reliable measure of recovery. For example the internal consistency is sufficient (Cronbach’s alpha is 0.86) and the concurrent validity is good (correlation with the Recovery Assessment Scale is 0.723) [20]. The I.ROC is preferred by service users when compared with other instruments [21], although one study [22] found the six-point response scale to be problematic.

Before the I.ROC could be used in the Netherlands, it needed to be translated and validated. The present study aimed, therefore, to (a) present the translation of the I.ROC from English to Dutch and (b) assess the psychometric properties of the Dutch version of the I.ROC when used with people with a severe mental illness who were receiving low-intensity community mental healthcare.

## Methods

This psychometric evaluation of the I.ROC was designed as a multi-center cohort study of mental-health service users with a severe mental illness. Aim of the study was to evaluate test–retest reliability, internal consistency, construct validity, convergent validity and discriminant validity. Participants of the main study completed the I.ROC and additional questionnaires three times, with a three-month interval between each testing. Additionally, data were collected from routine measurements from one of the involved mental healthcare services; however, these participants completed only two of the questionnaires (I.ROC and OQ-45). The study is reported in accordance with the STROBE guidelines [23], and the criteria used in the statistical analysis are those that Terwee et al. [24] proposed.

As described earlier, the I.ROC was developed to measure personal recovery in a practical and user-friendly way. In order to improve service users’ experience while completing the questionnaire, the tool was illustrated in a colorful and easy-to-read layout. The original I.ROC has four clusters (each in a different color), each of which has three questions on a page. The questions are formulated to help service users evaluate their experiences during the past three months. As in the original English version, every question is accompanied by 8-to-12 key words to clarify the question, and each question is accompanied by a six-point Likert scale on which the response options are *never*, *almost never*, *sometimes*, *often*, *most of the time*, and *all the time*. The first cluster is *home*, which includes questions about *mental health*, *life skills*, and *safety and comfort*. The second cluster is *opportunities*, which includes questions about *physical health*, *exercise and activity*, and *purpose and direction*. The third cluster is *other people*, which includes questions about *personal network*, *social network*, and *valuing myself*. The fourth and last cluster is *empowerment*, which includes questions about *participation and control*, *self-management*, and *hope for the future* [20]. The results from the administration of the questionnaire were plotted on a spider diagram for (a) ease of interpretation, (b) easy comparison with the previous administration of the questionnaire, and (c) facilitating the discussion of the results with the service user. The total mean score is calculated from all twelve items.

### Translation

The I.ROC was translated using a method that made use of committees, focus groups, and a back translation, as recommended by Epstein et al. [25]. The first translation from English to Dutch was done by authors BT and TB, in collaboration with a committee of nine healthcare professionals, researchers, and peer workers, all of whom were familiar with the care of people with a severe mental illness. In the next step, twelve service users were asked to review the translation, both individually and in a focus group. During the next stage, an independent professional translator back translated the Dutch version into English, and the back translation was then compared with the original English version. Where differences occurred, they were resolved in collaboration with the translators, the original developers of the I.ROC, and the committee that assisted in the first translation. Finally, the Dutch translation was transformed using the original layout of the I.ROC by the original artist, thereby creating a Dutch version that was comparable in appearance to the original English version.

### Setting

Participants were recruited from two primary mental healthcare services at six different locations, which spanned both regional cities and more rural areas. All adults who were receiving care for a severe mental illness at one of these services were invited to participate in the study. No characteristics were collected from the patients that did not participate. In order to receive this care for severe mental illness, the participants had to be diagnosed with any mental illness (although usually mood, anxiety, or personality disorder or to lesser extend a psychotic disorder), have problems in multiple areas of their life and need care from mental healthcare professionals for more than one year. Potential participants under 18 years old were excluded, as were participants who were unable to read Dutch.

### Data collection

On three occasions, with a three-month interval between each one, participants were asked by email to complete several digitized questionnaires on a secure website. Most participants needed between twenty and forty minutes to complete all questionnaires. Participants were told they could complete the questionnaires on different instances if they got tired, but were asked to complete the questionnaires within two weeks.

In order to assess test–retest reliability, a small number of additional participants were recruited from two locations in one primary mental healthcare service. The same exclusion criteria were used as in the original administration of the questionnaires. Participants who completed this part of the assessment were asked to complete only the I.ROC twice, with an interval of two weeks between each completion. The two-week interval was chosen because most of the participants were not expected to experience a major change in their situation within a two-week period. A shorter interval than two weeks would, however, have increased the risk for a recall effect in as much as the I.ROC has only 12 questions. In addition to the main part of the study, we collected data from participants who the I.ROC as routine outcome measurements during their treatment.

### Instruments

To assess the convergent and discriminant validity of the I.ROC, five additional questionnaires were administered to the participants in the main study as follows:

There was a general questionnaire about participants’ demographic characteristics, including their age, sex, and primary diagnosis (as determined by the healthcare professionals who were involved). It was administered only at baseline; all of the other questionnaires were administered at each of the assessment points.

The Manchester Short Assessment of quality of life (MANSA) is a quality-of-life measure which comprises 12 questions [26]. The MANSA has been shown to have good psychometric properties and has been used regularly in research studies, internationally and in the Netherlands [27]. Respondents’ answers are summed to yield a single total score.

The Recovery Assessment Scale (RAS) is the most frequently used questionnaire for measuring personal recovery [18]. It has been shown to have good psychometric properties [28]. Several formats of varying length have been proposed; in this study we used the original version, which includes 41 questions. Questions are answered on a five-point Likert scale, ranging from *1* (*Strongly Disagree*) to *5* (*Strongly Agree*). Individual questions are summed to give a total score, and five sub-scale scores for the factors called: *personal confidence and hope*, *goal and success orientation*, *willingness to ask for help*, *reliance on others*, and *no domination by symptoms* [29].

A single question, which was adapted from the Pharmacotherapy Monitoring and Outcome Survey (PHAMOUS) [30], was used to measure on a seven-point Likert-scale the amount of physical exercise that participants engaged in. The PHAMOUS was chosen because we needed to measure physical exercise in order to validate the I.ROC question about physical exercise, which is not included in any of the other instruments in the test battery. There is however a difference between both questions. The exercise question from PHAMOUS is about the number of days per week where the participant is physically active (moderate-intensively; increased heart-rate, getting warm), the physical exercise question from the I.ROC asks the participant to judge their own level of physical activity.

The final questionnaire was the Outcome Questionnaire (OQ-45), which is an instrument that has been frequently used both clinically and in scientific research [31] to measure symptoms of mental illness and social functioning and was selected for this study because the Dutch version is extensively researched and validated [32]. The OQ-45 has 45 questions that are answered on a 5-point Likert scale. Responses are summed to yield a single total score and scores on three sub-scales: *interpersonal relationships*, *social roles*, and *symptomatic distress*.

The participants included in the additional data collection from routine measurements completed only the I.ROC and the OQ-45 questionnaires. All questionnaires were completed by all participants as an online self-report without assistance.

### Analysis

All of the individual scores were checked to determine whether they were normally distributed and whether there were floor or ceiling effects by inspecting histograms of the scores. Means and standard deviations were then calculated for each of the individual items and the cluster scores. Test–retest reliability was evaluated by calculating the Intraclass Correlation Coefficient (ICC).

#### Internal consistency

In order to evaluate internal consistency, Cronbach’s alpha was calculated using the baseline measures for all of the participants, including the participants who were in the test–retest sample.

#### Factor analysis

An exploratory factor analysis was performed on the results from the I.ROC (limited to one observation per participant), using principal component analysis without rotation. The number of factors was determined by using the criterion eigenvalue > 1, and it was confirmed by inspecting the scree plot.

#### Convergent validity

In order to assess convergent validity, relationships among (a) the mean score or the I.ROC or an individual item of the I.ROC, and (b) the relevant scores from the other questionnaires (as determined before the start of the study), Pearson correlation coefficients were calculated. All of the I.ROCs (except for those used in the test–retest analysis) were included. A correlation of ≥ 0.70 was used as the *gold standard* for identifying a cut-off score for assessing convergent validity [24]. To evaluate discriminant validity, I.ROC scores for the different age, sex, and diagnostic groups were compared using *t*-tests or analysis of variance (ANOVA).

#### Sensitivity to change

To evaluate sensitivity to change, the effect sizes for the repeated measures were calculated using Cohen’s *d* for the I.ROC and the OQ-45.

### Ethical considerations

The (mental) burden on patients was minimised by using available data as much as possible and by not including more patients than necessary in the additional data collection. The study was approved by the Independent Review Board Nijmegen (Application Number IRBN2015015) and was performed according to all relevant guidelines and regulations, including the declaration of Helsinki. All participants gave informed consent prior to their participation.

## Results

A total of 124 service users participated in the main part of the study. Loss-to-follow-up was 48% after three months (64 measures remained) and 72% after six months (32 measures remained). In total 220 measures were collected. The mean time between the assessments was 87.2 days (*sd* = 8.84). Sixty-six (53%) of the participants were female, and the mean age of the participants was 49.8 years (*sd* = 12.3). Additional participants (*n* = 25) were included in the test–retest data collection; all of these participants completed two measurements. In addition to the main part of the study, we collected data from 615 participants who completed a total of 2,593 measurements with the I.ROC as routine outcome measurements during their treatment. Along with these measurements with the I.ROC, a simultaneous measurement with the OQ-45 was obtained on 1,440 occasions. See Table 1 for all collected measurements and information on which data was used for each analysis.

**Table 1:** Data use per statistical test

	Main study	Additional test–retest data	Routine Outcome Measurement data	TOTAL
Total available data	124 participants with 220 measurements	25 participants with 50 measurements	615 participants with 2,593 measurements	764 participants with 2863 measurements
Participant characteristics	124 participants with 124 measurements (baseline)			
Descriptive statistics and normal distribution	124 participants with 220 measurements		615 participants with 2,593 measurements	739 participants with 2813 measurements
Test–retest reliability	14 participants with 28 measurements	25 participants with 50 measurements		39 participants with 78 measurements
Internal consistency	124 participants with 124 measurements (baseline)		615 participants with 615 measurements (baseline)	739 participants with 739 measurements
Factor analysis	124 participants with 124 measurements		615 participants with 615 measurements	739 participants with 739 measurements
Convergent and divergent validity	124 participants with 127–217 measurements (due to missing data)			124 participants with 127–217 measurements (due to missing data)
Discrimintant validity	124 participants with 124 measurements (baseline)			
Sensitivity to change	64 participants with 128 measurements (after three months) 32 participants with 64 measurements (after six months)		291 participants with 582 measurements (after three months) 156 participants with 312 measurements (after six months)	255 participants with 710 measurements (after three months) 188 participants with 376 measurements (after six months)

The most common diagnoses for the sample were major depressive disorder and anxiety disorder. For additional information about the participants’ characteristics, see Table 2

**Table 2:** Participants’ characteristics (sex, age, and primary diagnosis) (*N* = 124)

Variables	*N* (%) or Mean (*sd*)
Number of women	66 (53%)
Age	49.8 (*sd* = 12.3)
Major depressive disorder	48 (38.7%)
Anxiety disorder	32 (25.8%)
Personality disorder	21 (16.9%)
Schizophrenia or another psychotic disorder	9 (7.3%)
Developmental disorder	8 (6.5%)
Bipolar mood disorder	3 (2.4%)
Another primary diagnosis	3 (2.4%)

Thirty-nine service users participated in the test–retest study, 14 of whom also participated in the main study and 25 who only participated in the test–retest study. The 39 participants in the test–retest study completed a total of 78 assessments. The mean time between the assessments was 13.7 days (*sd* = 6.93). Twenty-two (56%) of the participants who were included in the test–retest analysis were female; their mean age was 41.2 years (*sd* = 17.6).

### Descriptive statistics

All of the individual and mean scores were normally distributed (*n* = 2,813).

### Reliability

The Intraclass Correlation Coefficient (ICC) from the test–retest analysis was 0.856 (*n* = 39). The ICC is above the cutoff score of 0.70 [22], which indicates that the test–retest reliability was excellent.

Cronbach’s alpha for the I.ROC was 0.921 (*n* = 739), which is above the upper limit criterion of 0.90 that Terwee et al. [22] suggested. This implies that the I.ROC has high internal consistency, but it could probably measure the same construct using fewer variables.

### Validity

In the exploratory factor analysis (*n* = 739), when an eigenvalue of > 1 was used, it was obvious that a one-factor solution was appropriate for evaluating I.ROC’s construct validity. This was confirmed by inspecting the scree plot, in which there was a clear demarcation between the first factor and the remainder of the graph. All of the items had a loading greater than 0.5 on this factor.

For assessing convergent and divergent validity, correlations between the items on the I.ROC and the comparable scores and subscale scores from the other validated questionnaires were calculated. The results are displayed in Table 3, which shows that most of the items on the I.ROC were significantly correlated with the relevant scores and subscale scores from the other questionnaires, with the correlation coefficients ranging from 0.4 to 0.9. Some of the correlations were negative, indicating that there is a relationship between the scores, but that one score increases when the other decreases, for example when the symptoms of mental illness (measured with the OQ-45) increase, the personal recovery (measured with the I.ROC) decreases (-0.93, *p* < 0.001).

**Table 3:** Correlations between items from the I.ROC and the scale and subscale scores from the OQ-45, MANSA, and RAS (only data from main study used)

I.ROC **Items**	**OQ-45** **(*N* = 127)**	**MANSA (*N* = 217)**	**RAS** **(*N* = 215)**	**Exercise question (*N* = 178)**
Mental health	Subscale: symptomatic distress -0.79*	0.78*		
Life skills	Subscale: social role -0.432*			
Safety & comfort		0.59*		
Physical health		0.60*		
Exercise & activity				0.48*
Purpose & direction			Subscale: goal and success oriented 0.64*	
Personal network	Subscale: interpersonal relationships -0.65*		Subscale: rely on others 0.62*	
Social network	Subscale: interpersonal relationships -0.47*			
Valuing myself			Subscale: personal confidence and hope 0.77*	
Participation & control		0.65*	Subscale: personal confidence and hope 0.60*	
Self-management		0.68*	Subscale: 0.69*	
Hope for the future		0.78*	Subscale: personal confidence and hope 0.79*	
Total I.ROC score	-0.83*	0.85*	0.79*	

*Note*: * = *p* < 0.001

Regarding discriminant validity, the female service users scored significantly higher than the male service users on the mean total I.ROC score (*p* = 0.039), but there were no significant differences between the different age groups or between the different diagnostic groups. It should be noted, however, that some groups had small *n*s (see Table 4).

**Table 4:** I.ROC scores for subgroups

Variable	Subgroups	*N*	Mean	*SD*	*p*
**Sex**	Male	58	3.49	1.04	0.039
	Female	66	3.86	0.96	
**Age (in years)**	< 30	10	4.13	0.88	0.530
	30–45	37	3.62	0.99	
	46–60	55	3.66	1.02	
	> 60	22	3.66	1.01	
**Primary diagnosis**	Schizophrenia or another psychotic disorder	9	4.14	0.91	0.183
	Major depressive disorder	48	3.81	1.14	
	Anxiety disorder	32	3.61	0.92	
	Personality disorder	21	3.36	0.71	
	Another primary diagnosis	12	3.63	0.95	
**Total**		124	3.69	0.99	

### Sensitivity to change

In total, 355 sets of measurements for both the I.ROC and the OQ-45 with a three-month interval between each set were identified (the mean interval between the measurements was 90 days). There were 188 sets of measurements that were identified as being approximately six months apart (the mean difference between the measurements was 173 days).

For the I.ROC and its subscales, the effect sizes after three months were between 0 and 0.10, and they increase to between 0.02 and 0.16 after six months (see Table 5 for all of the effect sizes). The effect sizes for the OQ-45 and its subscales were between -0.10 and -0.02 after three months, and they increased to between 0.02 and 0.17 after six months.

**Table 5:** Participants’ sensitivity to change as measured at the three-month follow-up

Questionnaire	Item / scale	*ES* (Cohen’s *d*)	
		3 months (*n* = 355)	6 months (*n* = 188)
**I.ROC**	Mental health	0.084	0.155
	Life skills	0.055	0.036
	Safety & comfort	0.104	0.032
	Physical health	0.056	0.126
	Exercise & activity	0.039	0.123
	Purpose & direction	0.013	0.061
	Personal network	0.078	0.140
	Social network	0.103	0.016
	Valuing myself	0.049	0.066
	Participation & control	0.000	0.017
	Self-management	0.055	0.134
	Hope for the future	0.041	0.123
	Home	0.099	0.088
	Opportunity	0.046	0.128
	People	0.097	0.091
	Empowerment	0.036	0.091
	Total score	0.078	0.113
**OQ-45**	Symptomatic distress	-0.106	0.173
	Interpersonal relationships	-0.023	0.043
	Social role	-0.033	0.019
	Total score	-0.102	0.153

## Discussion

The aim of this study was to determine the psychometric properties of the Dutch version of the I.ROC in service users receiving low-intensity community mental healthcare. The Dutch I.ROC showed excellent test–retest reliability and internal consistency. Exploratory factor analysis indicated that a single factor solution for the Dutch version of I.ROC was optimal. Convergent validity using measures of personal and clinical recovery and quality of life were also excellent since most of the items on the I.ROC were significantly correlated with the relevant scores and subscale scores from the other questionnaires. Discriminant validity was inconclusive, although differences between male and female service users were identified. Sensitivity to change was 75% at the item level, but it did not reach this level on the total score from the I.ROC.

Some of the correlations that were identified in the assessment of convergent validity are noteworthy. The I.ROC item *exercise and activity*, for example, correlated only 0.478 with the question about exercise, although a higher correlation was expected because the two items clearly overlap. The mean score on the I.ROC was correlated with scores on the OQ-45, the MANSA, and the RAS, and the correlations were higher than the cut-off score of ≥ 0.70; they ranged between 0.791 and 0.853. The correlation between the total score on the I.ROC and the total score on the OQ-45 was negative. This was expected because higher scores on the I.ROC indicate better recovery, but higher scores on the OQ-45 indicate a greater number of symptoms and problems. The correlation between the I.ROC and both the MANSA and the RAS were positive. This was also expected because higher total scores on all three of these questionnaires indicate better recovery.

The results from the exploratory factor analysis were different from those that were published initially. Specifically, in earlier research, a two-factor solution was reported, in which the two factors on the I.ROC were labeled *interpersonal* and *intrapersonal* [19]. The present study shows, by contrast, that the Dutch version of the I.ROC has only one factor. This provides support for unidimensional models, which depict personal recovery as a holistic concept. This viewpoint is discussed in the Introduction to the current article, which concludes that all items on the I.ROC are consistent with this unidimensional concept. These results are also consistent with the results from a Rasch analysis of the English version of the I.ROC [22]. For use in daily clinical practice, a single factor from the I.ROC is advantageous, in that it enables the I.ROC to provide clear-cut results that are easy to interpret and discuss with the service user.

In this study, female participants scored significantly higher than male participants on the total score from the I.ROC, and they also scored higher, although not significantly so, on the total score from the MANSA, the RAS, and the OQ-45. Significant sex differences have not been commonly found in the total score on the MANSA, the RAS, or the OQ-45 [27, 32, 33]; thus, the nonsignificant differences found in this study support earlier results. No significant differences were found with regard to age or primary diagnosis, although nonsignificant differences were found when different sub-groups of participants were compared. The process of personal recovery develops differently in people from different sub-groups [34–36]. Thus, the hypothesis that was being tested was that different sub-groups of participants would have different scores on the I.ROC. The statistical power of this study proved, however, to be inadequate to confirm or reject this hypothesis.

There was a limited effect size (Cohen’s *d* = 0.113) associated with the change in the total score on the I.ROC after six months, with comparable effect sizes for the OQ-45 and the subscales of both questionnaires. The finding that the I.ROC and the OQ-45 have similar sensitivity to change is somewhat promising, as the OQ-45 is known to have an adequate sensitivity to change in the population of people with severe mental illness [37]. However, it would, of course, be preferable if the I.ROC had better sensitivity to change than the OQ-45.

The sensitivity to change of the Dutch translation of the I.ROC is promising, as the sensitivity to change of the I.ROC is comparable to the sensitivity to change of the OQ-45, despite having only 12 questions instead of 45. The overall low sensitivity to change could be due to the population tested in this study. The participants were already receiving treatment aimed at reducing their symptoms before they participated in the study, and during the study they received treatment that was aimed at stabilising their symptoms. It is possible that there was little improvement in the condition of the participants during the six-months follow-up due to the low-intensity community mental healthcare the participants received. This hypothesis has some support in that the I.ROC has been found to be more sensitive to change in other populations [21, 38]. However, based on these results, we can only conclude that although sensitivity to change might be promising, further research certainly needs to be conducted.

### Strengths and limitations

The major strong point of this study was the rigorous procedure that was followed in the translation of the I.ROC into Dutch. It included all of the steps that professional translators advise, including translation by a committee and an independent back translation. Additionally, by incorporating existing data into the study, the strain on participants was minimised. The major weakness of this study was the six-month interval between measurements, which was shorter than optimal. The study population might have changed too little during the six-month period to adequately evaluate the I.ROC’s sensitivity to change. Also, although there are no indications for selection bias, selection bias cannot be ruled out since no characteristics were collected about the patients who were asked to participate, but did not participate.

## Conclusions

The Dutch version of the I.ROC appears to have adequate psychometric properties to warrant its use in daily practice. The I.ROC was designed to measure patients’ overall personal recovery, which both the factor analysis and the measure of convergent validity supported. The I.ROC’s test–retest reliability was satisfactory, as was its internal consistency. Sensitivity to change was small, but it was comparable to that of the OQ-45. This aspect of the I.ROC needs additional research, as does the instrument’s discriminant validity.

## Data Availability

The datasets generated and/or analysed during the current study are available in the figshare repository, https://doi.org/10.6084/m9.figshare.12554672

## Abbreviations

I.ROC: Individual Recovery Outcome Counter
MANSA: MANchester Short Assessment of quality of life
OQ-45: Outcome Questionnaire 45
PHAMOUS: Pharmacotherapy Monitoring and Outcome Survey
RAS: Recovery Assessment Scale
